# Data-driven integrated care pathways: Standardization of delivering patient-centered care

**DOI:** 10.3389/fmed.2022.883874

**Published:** 2022-08-25

**Authors:** Shasha Han, Libing Ma

**Affiliations:** ^1^Beijing International Center for Mathematical Research, Peking University, Beijing, China; ^2^School of Population Medicine and Public Health, Chinese Academy of Medical Sciences and Peking Union Medical College, Beijing, China; ^3^Department of Respiratory and Critical Care Medicine, The Affiliated Hospital of Guilin Medical University, Guilin, China

**Keywords:** integrated care pathways, patient-centered care, digital clinical pathway, data-driven, care standardization

## Abstract

Health care delivery in China is in transition from reactive and doctor-centered to preventative and patient-centered. The challenge for the reform is to account for the needs of unique individuals and local communities while ensuring efficiency and equity. This Viewpoint presents data-driven integrated care pathways as a potential solution to standardize patient-centered care delivery, highlighting five core aspects of the entire care journey for personalization by using real-time data and digital technology, and identifying three capabilities to support the uptake of data-driven design.

## Introduction

Health care delivery in China is in transition from reactive and doctor-centered to preventative and patient-centered (1, 2). The shift is owning to increasing recognition of patients' critical role in care delivery (3). However, the absence of a widely shared tradition of professionalism may hinder the transition to more efficient and regulatory care delivery. Without adequate input from patients and physicians, top-down supervision cannot best serve their needs or may cause patient dissatisfaction and physician burnout (4, 5). Increasing data sharing has the potential to meet the gap. Data sharing allows for streamlining knowledge-sharing activities and learning initiatives using industrial data-driven approaches, and managing and governing care delivery with digital technologies.

However, how to use the data to standardize patient-centered care poses a challenge. Digital care pathways built upon real-time data could be a good solution. Data-driven pathways could serve as intelligent decision tools to improve patient care experiences by targeting individuals with varying health needs and increasing their engagement in care delivery. Also, data-driven pathways could help to improve care access (6). By pushing institutions to streamline data-sharing activities, data-driven pathways will help to connect in-home care and internet-based health systems to clinical services, and are likely to increase care access and continuity (6). Moreover, data-driven pathways can in turn foster data-sharing behaviors. Health institutions that have benefited from them will be motivated to improve data sharing activities, pushing the sharing of expertise in the entire health industry.

In this Viewpoint, we propose data-driven integrated care pathways. We show how real-time data and advanced technology can help to standardize patient-centered care. We start with the care pathways in China today, then outline the data-driven approach. We identify five core aspects across the entire care journey to build data-based capabilities, and elaborate on a hypothetical patient's experience. Finally, we discuss collaborative efforts to move high-value care from drawing boards to patients' hands.

## Patients' care pathways in China today

Integrated care pathway, also known as clinical pathway (7, 8), was a concept borrowed from industrial quality management technologies (specifically, the lean methodology) (9), and has been utilized by the Ministry of Health in China to monitor care delivery in hospitals. Pathways on about 224 disease conditions have been released (10). A care pathway consists of a structured multidisciplinary care plan that details essential steps in the care of patients with a specific clinical problem. The standard pathways aim to reduce unwanted variations in care delivery, particularly in the absence of scientific merit for local variability in treatment processes (11).

However, there is a lack of specifics about each care pathway. The existing national pathways only consider drug selection and hospital length of stay, leaving emotional support, information sharing, and care continuity largely untouched (10). With a wide choice of drug groups and hospital length of stays, physicians are essentially left to design their care pathways from scratch (11). Chaotic and inefficient pathways could be generated under sustained pressure to cope with operational targets. Admission decisions are made based on clinicians' judgments, and patients could be classified into different clinical groups in different institutions (11, 12). Treatment processes that deviate from pathways are often ignored (11, 12).

Also, a holistic pathway design that connects the entire care journey is currently lacking. Since health resources in China are primarily concentrated in affluent cities (13), patients in remote and rural areas may travel long distances for treatments in tertiary hospitals located in cities, and this may contribute to high hospital congestion and long delay in admission and discharge. Lack of sufficient community and home care before admission and after discharge is probably one contributor (14).

Furthermore, the orientation to patient-centered care, which requires care delivery to be respectful of patient preferences and needs (15), has complicated the initiative efforts to care standardization, especially when lacking data support (16). For example, when seeking to treat similar lung diseases, a 70-year-old retiree who has multimorbidity conditions is likely to prefer different pathways from a 30-year-old concrete manufacturer who is exposed to occupational dust. The retiree may need complicated medications with drug-drug interactions that require consideration (17), and the concrete manufacturer may need a quick surgery and regular physiotherapy (18). Although many care delivery systems have started considering such patient incentives (19, 20), they tend to rely on *ad hoc* self-reported data. Consequently, the resulting care cannot fully meet patients' needs at the moment that matters. To deliver patient-centered care, using real-time patient data is a must.

Altogether, these observations highlight that a holistic data-driven approach to pathway design could be the key to achieving the right balance between standardization and personalization.

## Data-driven integrated care pathways

**Figure 1:**
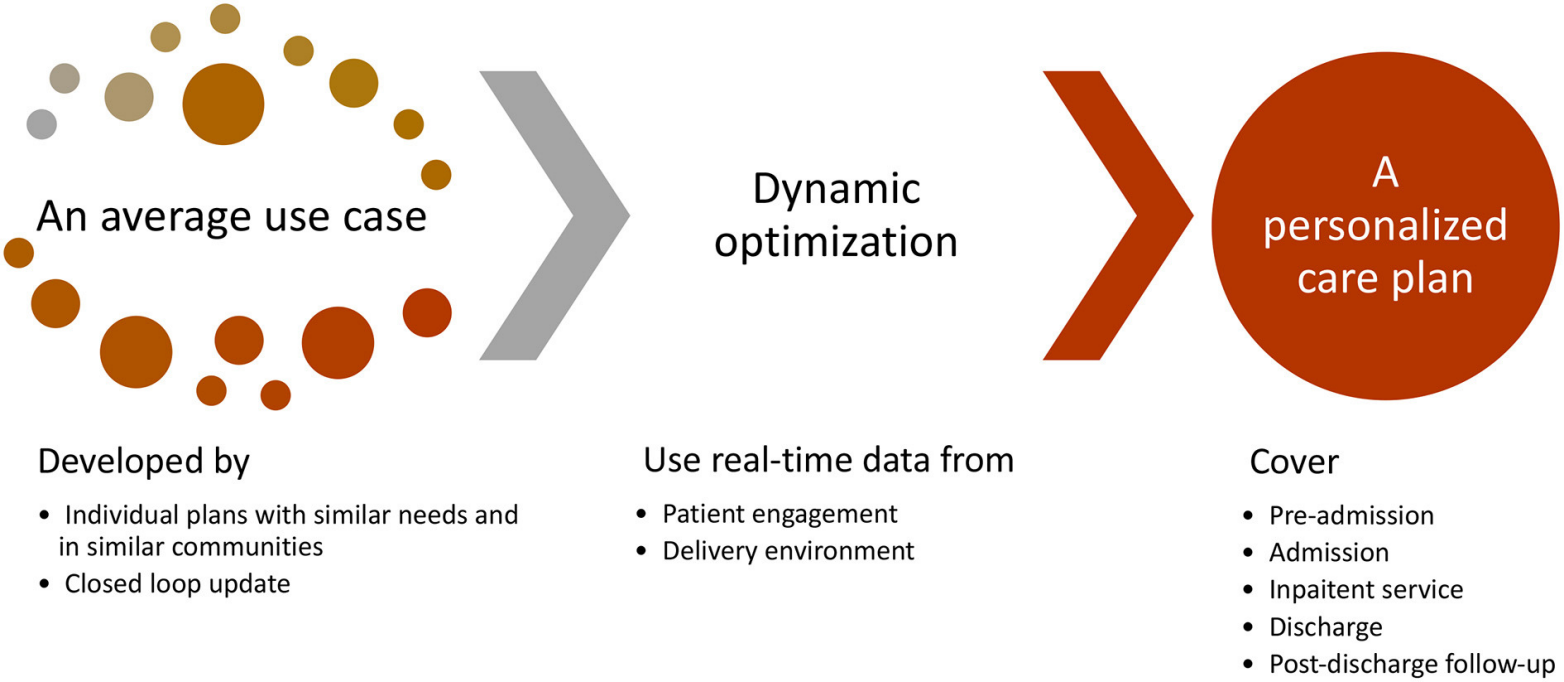
Data-driven integrated pathways.

Data-driven integrated care pathways (DICP) are digital tools to monitor care delivery using real-time data and advanced digital analytics. A data-driven integrated care pathway is a patient-centered care plan that incorporates appropriate variations from an average plan and supports shared decision-making with patients in the entire care journey (Figure 1). It consists of personalized operational specifications (e.g., who should do what, when, and where), and is updated promptly based on real-time feedback through digital technologies (e.g., artificial nurses, chatbot conversations). The goal is to explore adaptable pathways that can achieve desired health outcomes while reducing treatment costs. Constraints in optimization shall reflect the availability of medical resources and the time schedules of medical staff and patients.

DICP makes the balance between standardization and personalization through continuous improvement. On the one hand, the design dynamically optimizes care delivery using real-time data from patients and care providers. It uses dynamic optimization techniques to align quality-improvement drivers (e.g., bed utilization, patient discharge rates) from providers and the needs of patients, with average care plans serving as bases to increase model trackability in complex and dynamic clinical settings. On the other hand, the design continuously identifies opportunities for improving average plans. Important deviations from the current bases will signal personalization or the need to create new bases. Causal inference methods (21–23), optimization models (24–26), and machine learning techniques (27, 28) will be used to identify new standard and more adaptable pathways through nature experiments, computer simulations, and real-world data analysis (29, 30). Furthermore, DICP allows for close monitoring of personalized delivery processes, by checking the uniformity of average plans and personalization algorithms.

## Five core aspects of data-driven integrated care pathways

We have identified five aspects of the entire care journey in designing DICP, including pre-admission, admission, inpatient service, discharge, and post-discharge follow-up. Pathway design in China has traditionally focused on hospital-based services: admission, inpatient service, and discharge (10). However, we believe a seamless connection of inpatient service with pre-admission and post-discharge follow-up is critical to facilitate the disruptive transformation to a more preventative and patient-centered care delivery. We highlight that the data-driven design spans and connects the entire care journey with great engagement of patients, instead of restricting to inpatient care only. Various sources for patient data can be called upon, e.g., genomics, medical examination and laboratory test reports, primary care data, and third-party consumer data etc. In particular, real-time data collected from the ongoing care journey are required.

We show how data and technologies can be used to design DICP in the five aspects.

### Before admission, DICP employs applications and tools to monitor the health condition continuously

The task spans from real-time monitoring to early intervention and pre-diagnosis. First, DICP provides choices of self-care and enrollment alerts to patients using patient data, even when disease conditions are too mild to be noticed. Second, it empowers patients in lifestyle changes and early diagnosis through digital tools. Finally, it incorporates digital diagnosis tools into triage systems, including point-of-care diagnostics and emerging at-home tools, such as finger-prick home-testing kits, rapid response strep test kits, urinary tract infection tests, home thyroid TSH tests, and blood tests pressure monitoring. As such, the pre-admission phase of DICP allows individuals to monitor their health and educate themselves on preventative measures, which will help manage diseases before occurring and thus make the management more cost-efficient.

### During the admission phase, DICP helps identify suitable hospitals and proper pathways

In this phase, DICP will estimate expected costs and health outcomes for varying treatment timing and options using shared data from national, regional, and institutional levels. Also, DICP will predict higher beneficial care pathways and health institutions for an individual patient based on the patient's schedule, expected treatment costs, and desired health outcomes. Having patients select their preferred pathways based on health expectations and cost is likely to increase patient satisfaction with the treatment.

### During the inpatient service phase, DICP continuously adapts and refines treatment processes

DICP will adapt treatment processes with multimorbidity and comorbidity events, allowing for evidence-based treatment variability. Further, it will provide digital interventions (e.g., chatbot conversations) to improve information communication between providers and patients. In addition, it will refine care pathways based on the patients' real-time feedback and operational environment. As a consequence, DICP will provide an adaptative pathway. Such pathways are likely to help patients to be more involved in their care, learn about their condition and empower them to take control, which is central to delivering patient-centered care.

### During the discharge phase, DICP optimizes discharge processes

DICP refines the discharge from three aspects. First, it assists discharge processes and self-care after leaving hospitals through digital tools. Second, it employs mobile application videos to rehearse the self-care medications and exercises, the self-report on side-effects and adverse events, and the self-diagnosis with home-testing tools. Third, it enables a fast-track self-payment system as adopted by digitalized hotels. Such discharge process refining would create operational efficiencies and enhance patient and hospital staff experience.

### After patient discharge, DICP focuses on enhancing post-discharge follow-up and promoting recovery

In this phase, DICP will keep monitoring patient compliance and emotional healing through digital services such as chatbot conversations, virtual nurses, and online forum discussions. DICP will also develop alert systems to identify adverse events, disease relapse, and secondary complications. Coordinating with digital health services, e.g., offline hospitals, can help to reduce unnecessary follow-up visits.

For all these five aspects, various patient and operational data sources are required to make care delivery truly patient-centered. Moreover, strategic links between them are needed to enable the implementation of DICP.

## Envision patients' care experiences under DICP

**Figure 2:**
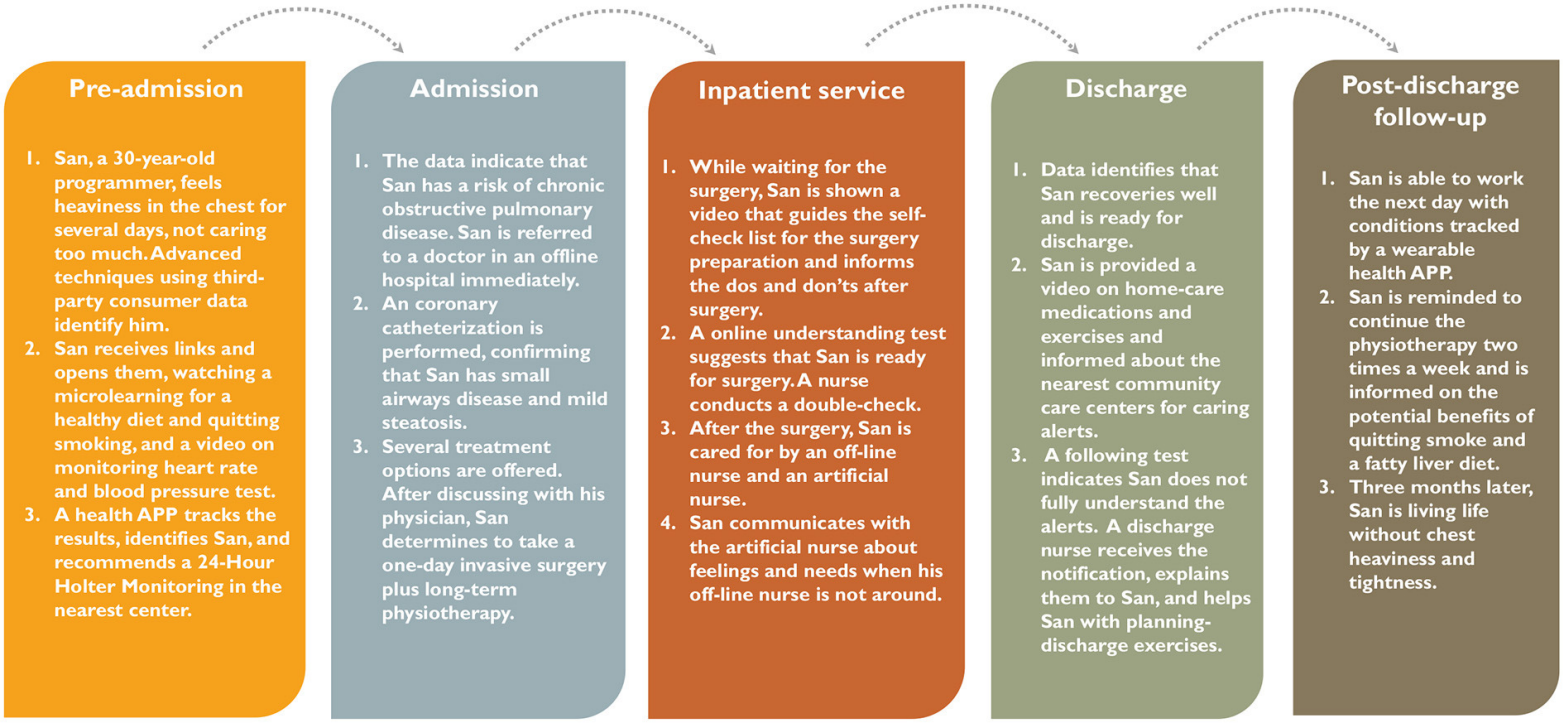
An example of patient experience under data-driven integrated care pathways.

We envision a hypothetical example of a patient's care process under DICP (Figure 2). In the example, the patient San experiences a streamlined care process, from pre-admission, admission, and inpatient services to discharge and post-discharge follow-ups, with real-time data and advanced technology being employed to improve his care experiences. First, DICP considers San's preferences and needs, helps him better understand his treatment options, and guides him to choose suitable care facilities and high-value pathways. Second, DICP employs digital technologies to increase San's engagement in the care process, providing emotional support to San when the nurses and physicians are not around, reminding San to comply with drugs and therapies, and helping San to prepare for discharge processes. Finally, DICP integrates community and home care with inpatient care, identifying San's abnormal conditions before the symptoms become severe, offering early diagnosis to San before admission, and monitoring San's compliance and emotional healing after discharge.

In addition, we observe that DICP could offer potential benefits to hospitals. While providing patient centered-care for San, DICP helps to decrease physicians' and nurses' cognitive workload, share time-consuming tasks (e.g., planning for discharge), and mitigate hospital congestions, which can lead to efficient resource use, few medical errors, and optimal staff scheduling.

## Discussions

Health institutions have yet to realize the full potential of data-driven integrated care pathways. By empowering providers and patients in care decisions, DICP design will help identify more patient-centered pathways. Moreover, DICP design provides a dynamic solution to improve health system operations using real-world data and advanced technologies. To amplify the value of DICP, we highlight that a holistic approach to building and strengthening the following capabilities is needed.

### Strengthen common digital health infrastructures

Standard digital health infrastructures enable individual applications and systems to interoperate and work in an integrated manner, serving as instrumental in supporting the process of data sharing. Therefore, to facilitate the uptake of DICP, institutions need to work on standardizing digital infrastructures. Some health institutions have already initiated electronic prescribing and ordering systems, electronic communication systems, clinical decision support tools, etc. (31). For them, the focus would be on advancing existing electronic sites and creating strategic linkages. Such institutions might need to integrate and optimize these primary care assets better and take initiatives in translating genomic and laboratory assets into clinical applications. For institutions in earlier stages of digital maturity, the focus would be to lay down an initial foundation on which future collaboration could be built. In addition, to enable a cohesive health platform among these institutions, system-wide common approaches such as WHO's digital health platform handbook could be adopted as guidelines in design and development (32).

Furthermore, strategic links between data from different systems need to be well-designed to balance the needs for data collection and management. We could employ novel technologies such as non-relational databases, distributed parallel computing, and deep data mining to address the problems of data storage (33). Nevertheless, even with the best design in place, a lack of accountability will thwart improvement. As such, a public sector is needed to initiate and supervise data-sharing activities, and monitor the personalized delivery processes. Moreover, data regulatory and security protection systems need to be improved to maximize the value of data while ensuring data security and personal privacy (34, 35).

### Enhance an integrated framework with advanced methods and techniques

The data-driven pathway is designed for real-time decision-making in complex and dynamic clinical settings and home care. To realize it, various sources of real-time data on patient data operation environments are required. Although hospitals in China have started investing in digital management efforts (31), most of them still lack the needed capabilities in data sharing and advanced analytics at scale. For example, real-time diagnostic and treatment information is not shared among hospitals, nor connected to the medical examinations in local healthcare centers (36). Nevertheless, real-time data sharing is crucial for the uptake of DICP. In real-timing data sharing, electronic medical records from hospitals and health digital tools would be timely updated into a central platform, where they are further linked to personal health data, including genomic and laboratory data, and surveillance data from public health services by data fusion techniques. Furthermore, advanced techniques for continuous data-sharing processes are needed to maintain the stability of sharing.

DICP serves as a real-time decision tool to make pathway improvement an integral and daily part of healthcare. Causal inference methods, optimization models, and machine learning techniques can be used to identify new standard and more adaptable pathways through nature experiments, computer simulations, and real-world data analysis (29, 30). Although adaptable pathways may bring concerns on upcoding of additional diagnoses to increase patient paying, cherry-picking of less costly patients, and even dumping unprofitable patients (37), these unintended consequences could be avoided or reduced by increasing real-time data sharing and adopting advanced inference techniques.

### Embed DICP implementation with existing systems and governance

Establishing these capabilities requires a focused effort within institutions, typically led by a single operation unit with support from executives and staff in other departments, as well as a coordinated effort between institutions at the local, regional, and national levels. Moreover, integrating such digital collaboration systems requires local and national government support and initiatives, including developing administrative capacity for platform management and supervision, strengthening data sharing rules and data security, and providing financial support and manpower resources.

A key place to start setting up digital collaboration systems could be within existing e-health governance and initiatives. From this perspective, we identify two factors for encouraging uptake of DICP. One is to rely on piloting medical partnerships such as healthcare consortiums and ecosystems to draw pooled resources of patient data (e.g., genomics, laboratory, and primary care data). The other is to increase the alignment of DICP with ongoing technological interventions, such as integrating them with the drug-related group-based payment and reimbursement system to accommodate patients' economic needs. Moreover, legislative and funding stimulus packages could be promising interventions to incentivize institutions to implement DICP collaboratively.

Once these capabilities and supportive tools are established, the integrated digitalized health systems will help to develop adaptable pathways. Collectively, the data-driven integrated care pathways, when applied broadly, will be able to make patient-centered care a reality in standard delivery.

## Author contributions

All authors listed have made a substantial, direct, and intellectual contribution to the work and approved it for publication.

## Funding

This study was supported by the Discipline Construction Funds of Population Medicine from Peking Union Medical College (No. WH10022021145) and the Talent Mini-highland Scientific Research Project of Guilin (No. 2020 3-05).

## Conflict of interest

The authors declare that the research was conducted in the absence of any commercial or financial relationships that could be construed as a potential conflict of interest.

## Publisher's note

All claims expressed in this article are solely those of the authors and do not necessarily represent those of their affiliated organizations, or those of the publisher, the editors and the reviewers. Any product that may be evaluated in this article, or claim that may be made by its manufacturer, is not guaranteed or endorsed by the publisher.
